# Cannabidiol enhances morphine antinociception, diminishes NMDA-mediated seizures and reduces stroke damage via the sigma 1 receptor

**DOI:** 10.1186/s13041-018-0395-2

**Published:** 2018-09-17

**Authors:** María Rodríguez-Muñoz, Yara Onetti, Elsa Cortés-Montero, Javier Garzón, Pilar Sánchez-Blázquez

**Affiliations:** Neuropharmacology. Department of Traslational Neuroscience, Cajal Institute, CSIC, E-28002 Madrid, Spain

**Keywords:** Cannabidiol, Cannabinoids, Sigma 1 receptor, NMDA receptor, Neuropathology, Epilepsy, Acute pain, Stroke

## Abstract

Cannabidiol (CBD), the major non-psychotomimetic compound present in the *Cannabis sativa* plant, exhibits therapeutic potential for various human diseases, including chronic neurodegenerative diseases, such as Alzheimer’s and Parkinson’s, ischemic stroke, epilepsy and other convulsive syndromes, neuropsychiatric disorders, neuropathic allodynia and certain types of cancer. CBD does not bind directly to endocannabinoid receptors 1 and 2, and despite research efforts, its specific targets remain to be fully identified. Notably, sigma 1 receptor (σ1R) antagonists inhibit glutamate *N*-methyl-D-aspartate acid receptor (NMDAR) activity and display positive effects on most of the aforesaid diseases. Thus, we investigated the effects of CBD on three animal models in which NMDAR overactivity plays a critical role: opioid analgesia attenuation, NMDA-induced convulsive syndrome and ischemic stroke. In an in vitro assay, CBD disrupted the regulatory association of σ1R with the NR1 subunit of NMDAR, an effect shared by σ1R antagonists, such as BD1063 and progesterone, and prevented by σ1R agonists, such as 4-IBP, PPCC and PRE084. The in vivo administration of CBD or BD1063 enhanced morphine-evoked supraspinal antinociception, alleviated NMDA-induced convulsive syndrome, and reduced the infarct size caused by permanent unilateral middle cerebral artery occlusion. These positive effects of CBD were reduced by the σ1R agonists PRE084 and PPCC, and absent in σ1R^−/−^ mice. Thus, CBD displays antagonist-like activity toward σ1R to reduce the negative effects of NMDAR overactivity in the abovementioned experimental situations.

## Introduction

Cannabidiol (CBD), a phytocannabinoid devoid of psychoactive properties, is currently being investigated in a series of clinical trials to determine its potential for treating diseases such as epilepsy, neuropsychiatric disorders, neurodegeneration and neuropathic allodynia [1–4]. The complete pharmacology of CBD is far from understood, as multiple mechanisms of action and several pharmacological effects have been proposed. Unlike Δ^9^-tetrahydrocannabinol, the main psychoactive constituent of the marijuana plant, the effects of CBD do not involve direct binding to the endocannabinoid receptors CB1 and CB2 [5, 6]; instead, CBD behaves as a non-competitive negative allosteric modulator of CB1 receptors [7]. Allosteric modulation, in conjunction with effects not mediated by CB1 receptors, may explain the in vivo effects of this compound. Indeed, CBD at nanomolar to micromolar concentrations is reported to interact with several non-endocannabinoid signaling systems to impair the function of orphan G-protein-coupled receptor (GPR) 55 [8], the transient receptor potential of melastatin type 8 channel and, transient receptor potential of ankyrin type 1 channel [9] and to facilitate the activity of serotonin 5HT1A receptor [10] and α3 and α1 glycine receptors [11, 12].

Within this complex framework, CBD exhibits positive effects in situations in which glutamatergic signaling, particularly that mediated by *N*-methyl-D-aspartate acid receptor (NMDAR), plays a critical role. Thus, CBD exhibits antioxidant properties and protects neurons from glutamate-induced death but without cannabinoid receptor activation or NMDAR antagonism [13]. CBD diminishes the neural damage caused by ischemic stroke [14] and chronic diseases, including Parkinson’s and Alzheimer’s diseases [15–17]. CBD also shows anticonvulsant activity in many acute animal models of seizures [18, 19], and in preclinical studies, CBD is comparable to antiepileptic drugs currently used in clinical therapy [20]. CBD also modulates morphine antinociception in mice [21] and exhibits anti-allodynia effects in rodent models of neuropathy [22, 23]. Indeed, CBD prevents the onset of mechanical and thermal sensitivity induced by the taxane chemotherapeutic agent paclitaxel in a female mouse model of chemotherapy-induced peripheral neuropathy [24]. Clinical evidence suggests that CBD can be used to manage epilepsy in adults and children affected by refractory seizures and exhibits a favorable side effect profile [25].

Similar to CBD, recent work has revealed that sigma 1 receptor (σ1R) antagonism prevents GPCRs from enhancing the function of NMDARs, thereby reducing the cellular impact of excessive glutamatergic activity [26, 27]. Thus, in the aforementioned situations, σ1R ligands, particularly antagonists, exhibit potential for treating neurological diseases [28], substance abuse syndromes [29], and certain neuropsychiatric disorders [30] and may serve as adjuvants for opioid analgesia [31]. Accordingly, σ1R antagonists alleviate neuropathic allodynia and inflammatory hyperalgesia in animal models of pain involving NMDAR activation [32–34]. Additionally, σ1R ligands have been shown to enhance neuroplasticity and functional recovery following experimental stroke [35], a paradigm in which increased NMDAR activity plays a decisive role. The highly selective σ1R antagonist, S1RA, significantly reduces the cerebral infarct size and neurological deficits caused by permanent middle cerebral artery occlusion (pMCAO) [36]. Likewise, recent data suggest the involvement of σ1R in rare CNS diseases, such as Dravet syndrome [37], and the sigma ligand ANAVEX 2–73 shows potential for treating certain CNS disorders [38], including epilepsy [https://www.anavex.com/anavex-releases-promising-full-preclinical-epilepsy-data-at-antiepileptic-drug-trials-xiii-conference/].

We thus addressed whether this phytocannabinoid modulates glutamatergic NMDAR transmission via σ1R. Our study suggests that CBD displays antagonist activity toward σ1R to inhibit NMDAR function and effectively reduces its ability to dampen morphine-induced analgesia, promote NMDA-mediated convulsive syndrome and cause neuronal damage after pMCAO.

## Results

### CBD activity toward the σ1R-NR1 complex

To regulate NMDAR function, σ1R binds in a calcium-dependent manner to the cytosolic regulatory sequence of the NMDAR NR1 subunit but not to the NR2A subunit [27, 39]. The NMDAR NR1 subunit has only a single σ1R binding site [40], which is located on the same cytosolic region that binds calcium-activated calmodulin (CaM) to reduce the probability of calcium channel opening [41]. We have described an in vitro assay that analyzes the capacity of drugs to alter the interaction of recombinant σ1R with the regulatory cytosolic C0-C1-C2 region of the NMDAR NR1 subunit [42]. In binding assays performed using brain membranes or cells with forced σ1R expression, the affinity of ligands competing with labeled σ1R ligands is in the nM range, probably because tritiated tracers provide reliable specific signals through their binding to the most abundant low affinity state of the receptors. In the in vitro setting, σ1R antagonists at pM concentrations disrupt σ1R-NR1 associations in a concentration-dependent manner, and pM concentrations of agonists prevent the effects of the former [26, 27]. As observed for GPCRs coupled to G proteins, the affinity of σ1R when bound to target proteins increases; thus, σ1R ligands display pM instead of nM activity for σ1R-NR1 associations.

**Fig. 1:**
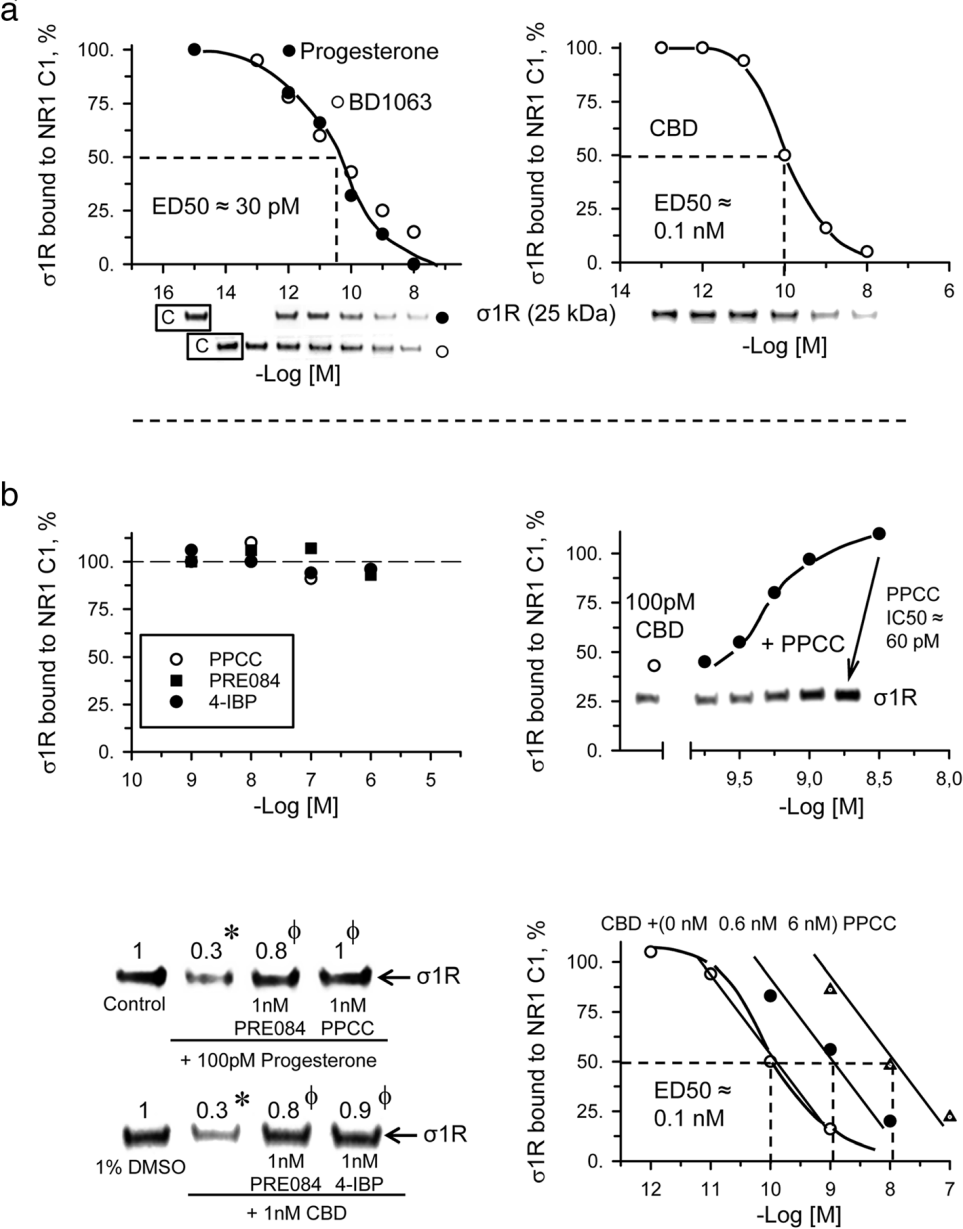
CBD disrupts the association of σ1R with the NR1 subunits of NMDA receptors. In vitro assay determining ligand activity for σ1R. NHS-activated Sepharose beads covalently coupled to a sequence of the NR1 subunit containing seven residues of the transmembrane region plus C0-C1-C2 cytosolic segments were incubated with excess σ1R (1:3). Unbound σ1R was washed out, and the NR1 C1-coupled σ1R was exposed to serial concentrations of the ligands under study. σ1R that remained attached to the NR1 subunits was then evaluated by SDS-PAGE and immunoblotting. **a** Inhibitory effects of the σ1R antagonists progesterone and BD106 and of CBD on the association of σ1R with the NR1 C1 subunit. The assays were performed in the presence of 50 mM Tris-HCl (pH 7.5), 0.2% CHAPS and 2.5 mM calcium. Representative blots are shown. The ED50 values were computed using the software SigmaPlot v.14. **b** The σ1R agonists PPCC, PRE084 and 4-IBP did not alter σ1R-NR1 associations but reduced the capacity of progesterone and CBD to disrupt such associations. PPCC reduced the capacity of CBD to inhibit the binding of σ1R to NR1 subunit in a concentration-dependent manner. The assays were performed twice, and each point was duplicated. *Significant difference with respect to the control group (DMSO or saline); φ significant difference with respect to the group receiving only CBD or progesterone; ANOVA, Dunnett’s multiple comparison, *p* < 0.05

The last transmembrane region plus the cytosolic C0-C1-C2 C-terminal sequence of the NR1 subunit was covalently attached to agarose particles (see Methods). Thus, agarose-NR1 was incubated with σ1R, and after removal of the unbound σ1R, the agarose-NR1-σ1R complexes were tested to determine the effects of potential σ1R ligands. Afterwards, the extent of σ1R binding to the agarose-NR1 subunits was subsequently evaluated. The endogenous neurosteroid progesterone and the synthetic drug BD1063, both putative σ1R antagonists, exhibited ED50s of approximately 30 pM for diminishing σ1R-NR1 complex formation (Fig. 1a). Other drugs displaying antagonist activity toward σ1R, such as S1RA, fenfluramine and norfenfluramine, also showed pM activity in this in vitro setting [42]. Notably, CBD displayed a dose-dependent capacity for diminishing σ1R-NR1 associations with an ED50 of approximately 100 pM; thus, CBD behaved as a σ1R antagonist (Fig. 1a). The σ1R agonists PRE084, 4-IBP and PPCC, which did not exhibit the capacity to disrupt σ1R-NR1 complexes, antagonized the inhibitory effect of progesterone and CBD on these complexes (Fig. 1b). PPCC reduced the activity of 100 pM CBD in a concentration-dependent manner with an apparent Ki of 60 pM. In the presence of increasing concentrations of PPCC, the curves of CBD (disrupting σ1R-NR1 associations) shifted to the right (Fig. 1b). Thus, recognized σ1R antagonists and CBD diminished the interaction of σ1R with the NR1 subunit of the NMDA receptor. These data suggest that CBD does bind to σ1R to trigger its antagonist effects and disrupt σ1R-NR1 regulatory interactions.

### CBD regulates morphine antinociception

The mu-opioid receptor in the mesencephalic periaqueductal gray matter plays the most relevant role in the antinociception produced by opioids injected by the icv route. The icv administration of all the substances studied circumvented the possibility that the drugs reached receptors beyond the brain. Subsequently, the capacity of morphine to produce supraspinal antinociception and the ability of the studied drugs to modulate this effect were studied through the warm water tail flick test.

**Fig. 2:**
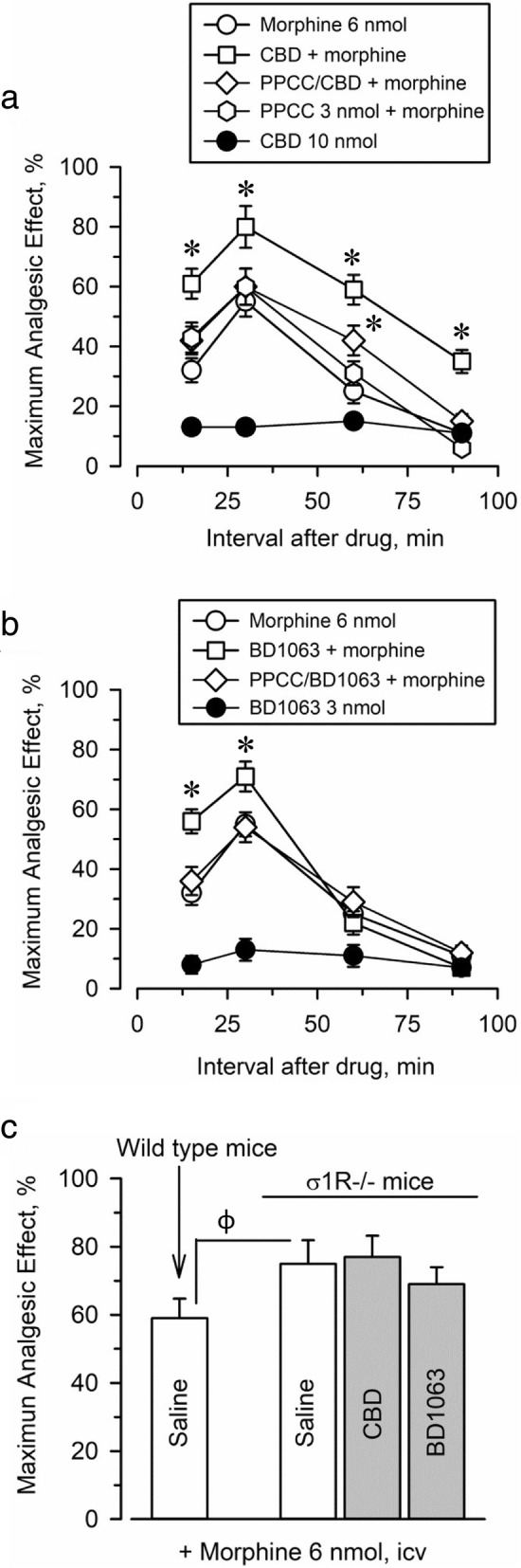
Effect of CBD on morphine-induced supraspinal antinociception. Mice received 10 nmol CBD icv 10 min before 6 nmol morphine, and analgesia was evaluated with the thermal warm water (52 °C) “tail-flick” test at the indicated post-opioid intervals. Each point represents the mean ± SEM of data from eight to ten mice. **a** CBD exhibited no significant analgesic effect in this test. The analgesia produced by the combination of CBD and morphine was significantly higher than that produced by morphine alone. The σ1R agonist PPCC did not alter morphine analgesia, but icv-injection 20 min before CBD prevented the enhancement of this effect of morphine. **b** The σ1R agonist BD1063 did not produce analgesia in this test but increased morphine antinociception. This potentiation was absent when PPCC was injected icv 20 min before BD1063. **c** Morphine promotes a higher analgesic effect in σ1R^−/−^ mice than in wild type control mice. In σ1R^−/−^ mice, BD1063 and CBD did not modify morphine analgesia. *Significantly different from the control group receiving only 6 nmol morphine, φ significantly different from the effect of morphine in wild-type mice. ANOVA, Dunnett’s multiple comparison vs control group, *p* < 0.05

The time-dependent antinociceptive effects of morphine, CBD, BD1063, PPCC, and combinations of these drugs were then investigated (Fig. 2). The analgesic effect of 6 nmol morphine icv peaked after 30 min reaching approximately 60% of the maximum analgesic effect measurable in this test. While CBD and the σ1R agonist PPCC did not promote antinociception in this assay, the administration of CBD (3 nmol, icv) before morphine treatment (6 nmol, icv) augmented the antinociceptive activity of the opioid (Fig. 2a). The maximum effect was observed when CBD was injected 10 min before the opioid, and this interval was used in subsequent experiments. Thus, in mice pretreated with CBD, morphine analgesia increased from 57.8 ± 4.3% to 83.4 ± 6.1% of the maximum analgesic effect.

It is known that S1RA and other σ1R antagonists administered icv at a low nmol range increase morphine analgesia in rodents, probably by the removal of a tonic constraint that this σ1R may exert over mu-opioid function [26, 27]. Indeed, pretreatment with 3 nmol BD1063 also potentiated morphine analgesia (from 55.0 ± 4.2% to 75.2 ± 5.1%) (Fig. 2b). The σ1R agonist PPCC did not affect morphine antinociception but prevented CBD and BD1063 from enhancing this effect of morphine (Fig. 2a and b). The involvement of σ1R in the effects of BD1063 and CBD was ascertained using sigma receptor knockout (σ1R^−/−^) mice. In agreement with previous reports [27], morphine yields higher analgesia in σ1R^−/−^ mice than in their wild-type control counterparts, with an approximately 35% increase at the peak interval of 30 min post-morphine. This effect of morphine could not be altered by BD1063 or CBD in the σ1R^−/−^ mice (Fig. 2c).

### Anticonvulsant activity of CBD

**Fig. 3:**
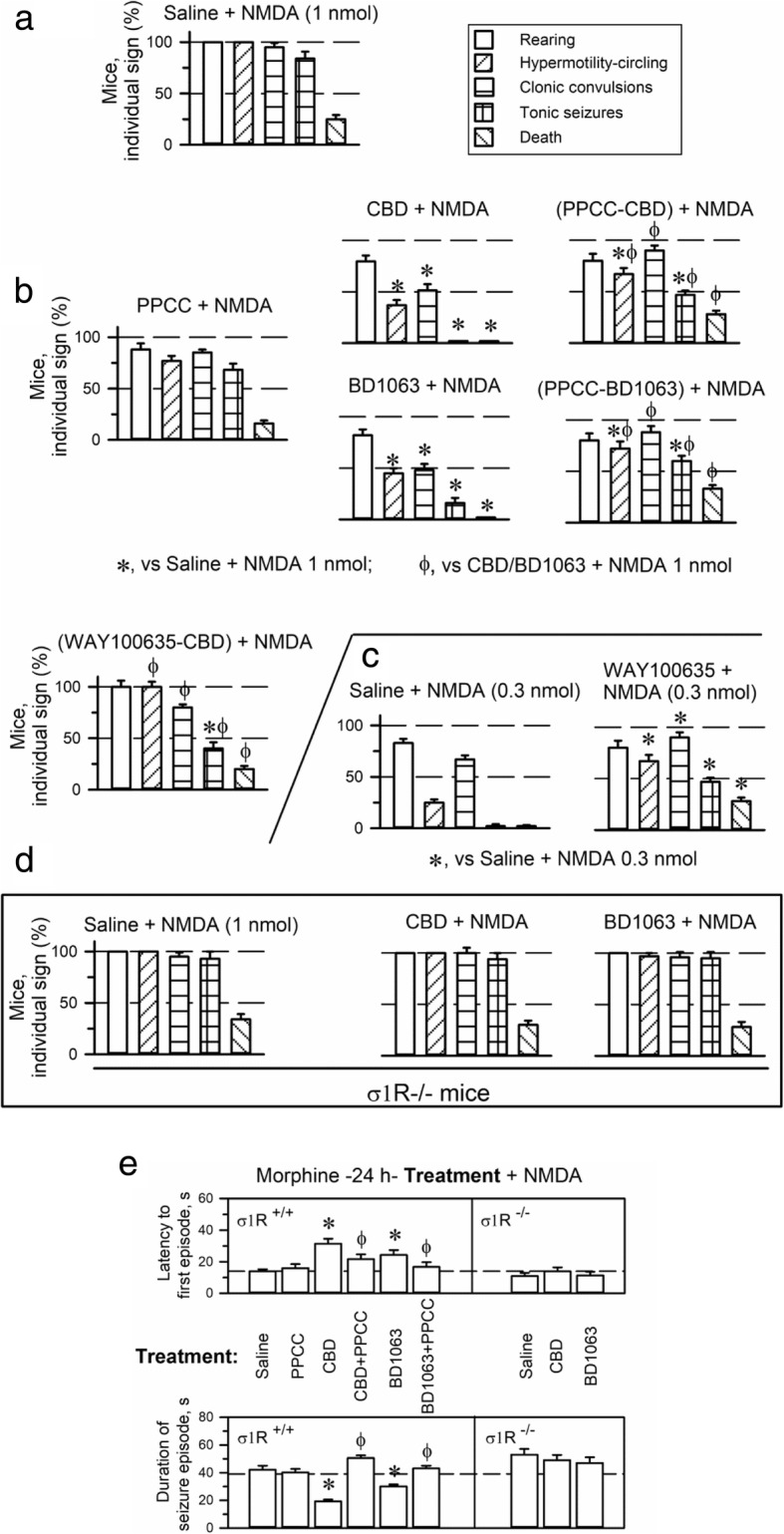
Anticonvulsant effects of CBD in a mouse model of seizures induced by NMDAR overactivation. **a** Behavioral alterations produced by the icv administration of 1 nmol NMDA to mice. Each bar indicates the percentage of mice showing the indicated sign and represents the mean ± SEM of 8 mice. **b** Effects of PPCC, CBD and BD1063 on seizures induced by NMDA. The mice received the NMDAR agonist NMDA icv (1 nmol) 30 min after the drugs (3 nmol PPCC, BD1063, CBD or 5 nmol WAY100635) and were then immediately evaluated. **c** Effect of the 5HT1AR antagonist WAY100635 on the convulsive syndrome evoked by 0.3 nmol NMDA. **d** Lack of an effect of CBD and BD1063 on seizures induced by 1 nmol NMDA in σ1R^−/−^ mice. **e** Effects of the treatments on the latency and duration of the seizure episodes induced by 1 nmol NMDA in wild type and σ1R^−/−^ mice. *Significant difference from the control group receiving NMDA and saline instead of the drugs. φ Significant difference from the corresponding NMDA-induced behavioral signs exhibited by the group receiving only CBD or BD1063, *p* < 0.05. ANOVA, Dunnett’s multiple comparison vs control group, *p* < 0.05

Selective σ1R antagonists diminish the manifestation of the NMDA-induced convulsive syndrome [42]. Thus, the anticonvulsant activity of CBD was evaluated in an animal model in which seizures were induced by the icv administration of NMDA [43, 44]. Indeed, a dose of 1 nmol NMDA icv induced tonic convulsions in approximately 95% of the mice. With this procedure, practically all the mice exhibited a series of anomalous behaviors, such as compulsive rearing, wild running (hypermobility and circling), clonic convulsions, tonic seizures, and, in approximately 15–20% of the animals, death (Fig. 3a). The σ1R agonist PPCC did not significantly alter the behavioral effects evoked by NMDA administration. In contrast, BD1063 and CBD protected more than 50% of the mice tested from hypermobility, convulsive rearing and clonic convulsions. Moreover, tonic seizures were present in only 20% of the mice, and no mice died. Notably, the σ1R agonist PPCC counteracted the ability of CBD and BD1063 to alleviate the NMDA-induced convulsive syndrome (Fig. 3b), indicating that σ1R is necessary for CBD to produce these beneficial effects.

It has been suggested that 5HT1AR participates in CBD activity [10]. In the presence of the 5HT1AR antagonist WAY100635, CBD exhibited a reduced capacity to diminish the convulsions promoted by 1 nmol NMDA. However, WAY100635 revealed an endogenous negative control of serotonin 5HT1AR on NMDAR overactivity. Thus, WAY100635 alone enhanced wild running and tonic seizures and promoted death in mice treated with the lower dose of 0.3 nmol NMDA (Fig. 3c). Notably, the positive effects of CBD and BD1063 on diminishing NMDA-induced convulsive signs were absent when the syndrome was modeled in σ1R^−/−^ mice (Fig. 3d).

While PPCC did not modify the latency to the first convulsive episode or its duration, CBD and BD1063 increased this latency and reduced the duration of the seizure episode. PPCC antagonized these effects of CBD and BD1063. In σ1R^−/−^ mice, the latency, duration and intensity of the NMDA-induced seizures were not altered by the administration of CBD or BD1063 (Fig. 3e).

### CBD diminished neural damage promoted by pMCAO

**Fig. 4:**
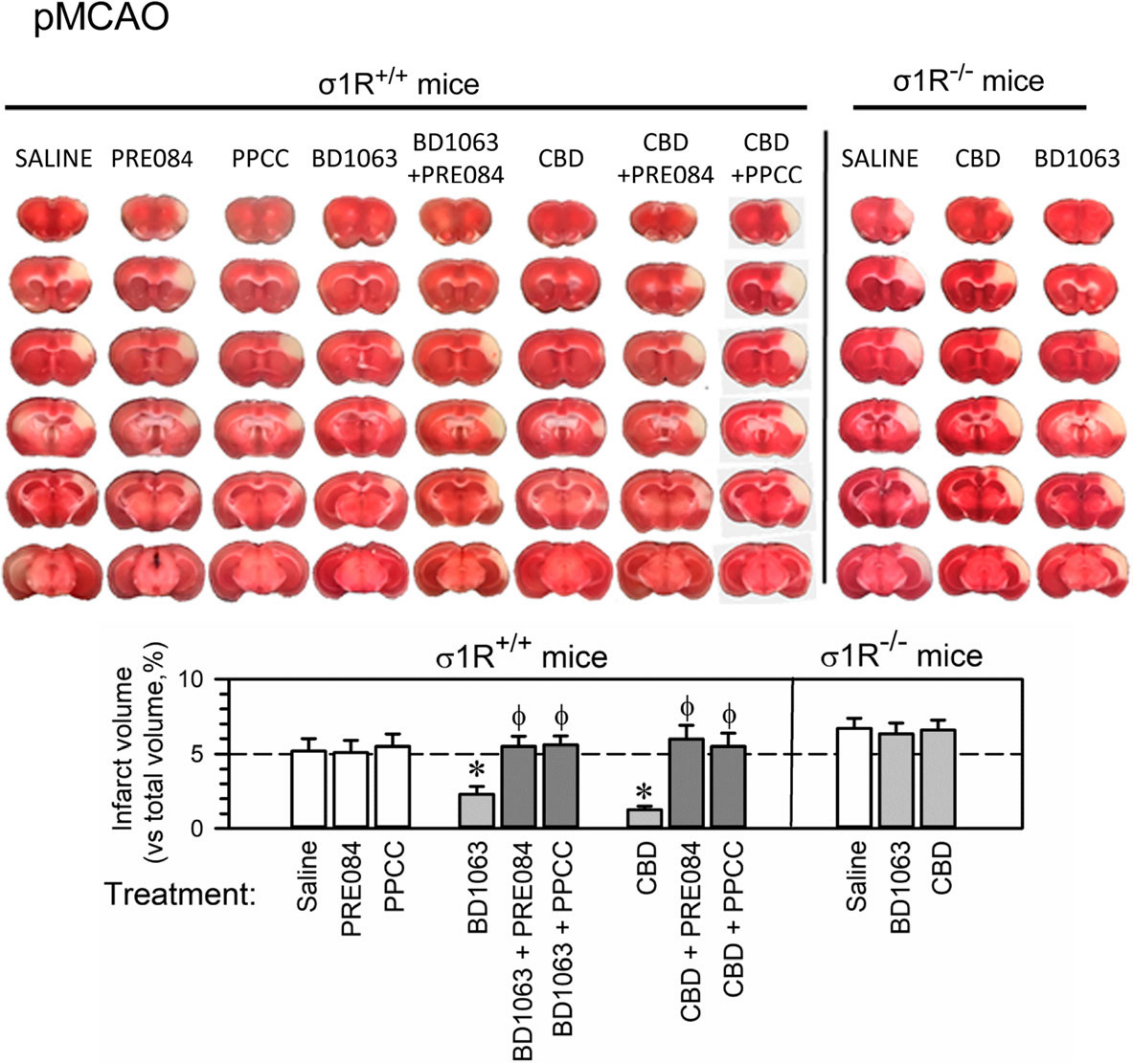
CBD administration diminishes ischemic brain damage in wild-type but not σ1R^−/−^ mice. Upper panel, representative TTC-stained brain section images obtained from saline- and drug-treated mice (1 h after surgery) 48 h after pMCAO. White indicates infarction; red staining indicates normal tissue. Lower panel, the bar graphs quantitatively compare the infarct volume based on TTC staining from the wild type and σ1R^−/−^ mice treated with saline, the σ1R agonists PRE084 and PPCC (white bars), or the σ1R antagonist BD1063 and CBD (gray bars) 1 h after surgery. The groups consisted of 8 to 10 mice, and the data are presented as the mean ± SEM. *Significantly different from the saline-treated mice. φ Significantly different from mice receiving only the σ1R antagonist BD1063 or CBD; ANOVA, Dunnett’s multiple comparison vs the corresponding control group, *p* < 0.05

The administration of CBD (10 nmol, icv) 60 min post-surgery resulted in much less severe infarction. The volumetric analysis of the brain showed that neither surgery nor the icv procedure significantly changed the total brain volume (302.9 ± 13.9 mm^3^ and 300.9 ± 7.9 mm^3^, respectively; sham-operated mice: 299.2 ± 5.9 mm^3^). However, pMCAO resulted in severe injury in mice examined 48 h after ischemia (Fig. 4). Injury was most apparent in the cerebral cortex, and the infarct volume was estimated to affect 5.4 ± 1.2% of the total brain volume. No damage was observed in the sham-operated mice. Compared with no treatment, the administration of CBD improved stroke outcomes (an approximate 75% reduction in the infarct size to 1.2 ± 0.9% of the total brain volume) after permanent cerebral ischemia. In agreement with previous reports [36], the selective σ1R antagonist BD1063 exhibited protective effects in this model. The σ1R agonist PRE084 does not alter the infarct volume promoted by pMCAO; however, PRE084 did prevent the neuroprotective effects of CBD and BD1063. As previously observed [36], σ1R^−/−^ mice showed increased infarct volumes (up 6.73 ± 1.8% of brain volume) with respect to their wild-type (σ1R^+/+^) controls, which were refractory to administration of either CBD or BD1063 (Fig. 4).

## Discussion

The present study shows that CBD acting as an antagonist of σ1R diminishes the influence of glutamate NMDA receptors in three experimental paradigms in which this activity plays a key role, i.e., the level of morphine-evoked antinociception, the incidence of NMDA-induced convulsive activity and the extent of the neural damage caused by experimental ictus. These positive effects of CBD are also achieved by the direct binding of drugs to the NMDAR ionic pore to block NMDAR calcium influx (antagonists) [45–47] and by antagonists, but not agonists, of σ1R, which regulates NMDAR function [36, 48, 49]. σ1R antagonists and CBD exhibit no such control over NMDAR activity in mice lacking σ1R protein expression. This and previous observations show that CBD does not bind to the NMDAR ionic pore [13] and thus suggest that CBD displays antagonist-like activity on σ1R to counteract the negative effects of NMDAR hyperfunction in the aforementioned experimental situations.

The activity of glutamate NMDARs falls under the negative influence of some GPCRs, including CB1R [50], acetylcholine type 1 muscarinic receptor [51], serotonin 5HT1AR [52], adrenergic α1R and α2R [53], dopamine D3R and D4R [54, 55], and group III mGluR7R [56]. Among these GPCRs, 5HT1AR is a suitable candidate for mediating CBD effects [10], and in fact, the 5HT1AR antagonist WAY100635 diminished the capacity of CBD to alleviate NMDA-induced convulsive syndrome. Nevertheless, WAY100635 alone enhanced the capacity of the agonist NMDA to evoke convulsions, suggesting the endogenous inhibitory control of NMDAR activity by 5HT1AR. Certainly, WAY100635 has been described to display agonist activity toward the dopamine D4 receptor, which is negatively coupled to glutamate activity [57]. In our experimental model, WAY100635 enhanced wild running and tonic seizures and promoted death in mice treated with a lower dose of 0.3 nmol NMDA. Thus, in our convulsive model, the activity of WAY100635 toward dopamine D4 receptors can be disregarded. If CBD displays activity toward certain GPCRs to alleviate NMDA-induced convulsions, these receptors require the presence of σ1R to promote such an effect because CBD failed to do so in σ1R^−/−^ mice. The σ1R agonist PPCC produced no significant changes in this model but efficaciously counteracted the anticonvulsant effects of CBD. These observations suggest an essential role of σ1R in the negative control that CBD exerts on NMDAR activity. Indeed, in the presence of σ1R agonists, CBD did not exhibit the aforementioned effects, and the in vitro assays showed that CBD acts as a σ1R antagonist to disrupt σ1R-NR1 complexes.

Although σ1R is a chaperone that regulates a series of signaling proteins in the endoplasmic reticulum in a calcium-dependent manner, this protein was discovered as a type of opioid receptor, and σ1R can thus be found in the cell plasma membrane [58]. In this context, σ1R is a regulator of NMDAR function and cooperates with histidine triad nucleotide-binding protein 1 (HINT1) to regulate NMDAR function via certain GPCRs [26]. The calcium-dependent binding of σ1R to NMDAR NR1 subunits that carry the cytosolic C1 segment protects the activity of NMDARs, i.e., calcium influx, from the inhibitory action of calcium-activated calmodulin (Ca^2+^-CaM). While agonists promote σ1R binding to the NMDAR NR1 subunit, antagonists such as CBD disrupt these complexes to facilitate the Ca^2+^-CaM inhibition of NMDAR function. Thus, CBD acts as a σ1R antagonist to reduce NMDAR activity.

As previously mentioned, CBD is involved in a variety of activities and may act as a sedative, anxiolytic, antipsychotic, anti-inflammatory, antioxidative, neuroprotective, anti-emetic, anticancer, antidepressant and mood-stabilizing drug, as well as have therapeutic effects on movement disorders, ischemia, diabetes, and cannabis withdrawal syndrome [30, 59–63]. Moreover, CBD exhibits anti-neuropathic effects [22, 23] and modulates morphine antinociception in mice [21]. Our data suggest that CBD acts on σ1R to alleviate the manifestation of epileptic syndrome, to protect against neural damage caused by vascular ischemia and to enhance the antinociception promoted by morphine. These findings suggest the implication of σ1R in other beneficial activities attributed to this phytocannabinoid. Indeed, σ1R is a potential target for the treatment of neuropathic pain because it interacts with and regulates NMDARs and TRPV1 calcium channels, which are key constituents of the mechanisms that modulate activity-induced sensitization in nociceptive pathways [32–34, 64]. Moreover, σ1R ligands also exhibit antidepressant, anxiolytic, neuroprotective and antioxidative effects [30, 65].

Alterations in σ1R have been consistently related to schizophrenia [66, 67]. NMDAR function is lower in this mental illness, and the negative control exerted by GPCRs, such as CB1Rs, on glutamate activity may play an essential role in the etiology of this disease [68–70]. The severity of the negative symptoms of schizophrenic patients correlates with alterations in the plasma levels of anandamide [71] and with those of neurosteroids, the putative ligands of this ligand-operated chaperone/receptor [72, 73]. Indeed, adjunct treatment with pregnenolone diminishes the negative symptoms of schizophrenia [74]. The idea that the CB1R localizes primarily in axon terminals have already been challenged [75], and a series of immunocytochemical and ultrastructural studies have demonstrated the presence of the CB1R in the somatodendritic compartment (post-synapse), both at the spinal and supraspinal levels where it co-localizes with NMDARs and PSD95 proteins. Thus, an anomalous σ1R-regulated connection between CB1R and NMDAR may contribute to the disproportionate downregulation of NMDAR activity (hypofunction), constituting a serious risk factor for the development of schizophrenia [68, 76]. In the context of negative NMDAR regulation by CB1Rs, σ1R antagonists such as CBD uncouple NMDAR function from the negative influence of GPCRs, such as CB1Rs [70, 76].

In the sub-micromolar and micromolar range, CBD affects the function of various signaling pathways. Among other targets, CBD regulates cannabinoid receptors without displaying binding directly to them; CBD also impairs the function of the equilibrative nucleoside transporter, that of the orphan GPR55 receptor and that of the transient receptor potential of melastatin type 8 channel [8, 9]. Conversely, CBD enhances 5HT1AR, α3 and α1 glycine receptor, and transient receptor potential of ankyrin type 1 channel activity [9]. In hippocampal cultures, the CBD-mediated regulation of calcium levels is bidirectional and depends on the excitability of the cells [77]. CBD also activates the nuclear peroxisome proliferator-activated receptor γ and transient receptor potential vanilloid type 1 and 2 channels while inhibiting the cellular uptake and fatty acid amide hydrolase-catalyzed degradation of anandamide [71, 78]. CBD reduces hydroperoxide-induced oxidative damage, tissue cyclooxygenase activity, the nitric oxide production, T-cell responses, bioactive tumor necrosis factor release, and prostaglandin E2, cytokine interferon c and tumor necrosis factor production and blocks voltage-gated Na + channels [6, 77]. Whether the effects of CBD on σ1R are relevant to these activities remains to be explored. Because this exogenous cannabinoid may alter the function of a wide variety of cellular activities, the key is to determine the molecular mechanisms that are primarily implicated in a particular effect of CBD.

Our study indicates that CBD regulates the function of σ1R in at least several of the abovementioned behavioral effects. Thus, CBD’s enhancement of opioid analgesia, alleviation of convulsive syndrome, protection against ischemic neural damage and anti-allodynia effects appear to involve an antagonist interaction with σ1R and the subsequent reduction of NMDAR function. This finding may help in us to understand the current pharmacology of CBD and provides new avenues for the treatment of several brain-related disorders.

## Methods

### Expression of recombinant proteins

The coding region of murine full-length (1–223) σ1R (AF004927), and the C-terminal region of the glutamate NMDAR NR1 subunit (NM_008169) (residues 827–938), were amplified by RT-PCR using total RNA isolated from mouse brains as the template. Specific primers containing an upstream Sgf I restriction site and a downstream Pme I restriction site were used, as described previously [27]. The PCR products were cloned downstream of the GST coding sequence and the TEV protease site. The sequenced proteins were identical to the GenBank™ sequences. The vector was introduced into *E. coli* BL21 (KRX #L3002, Promega, Madrid, Spain), and clones were selected on solid medium containing ampicillin. After 3 h of induction at room temperature (1 mM IPTG and 0.1% Rhamnose), the cells were collected by centrifugation, and the pellets were maintained at − 80 °C. The GST fusion proteins were purified under native conditions on GStrap FF columns (GE#17–5130-01, Healthcare, Barcelona, Spain); when necessary, the fusion proteins retained were cleaved on the column with ProTEV protease (Promega, #V605A) and further purification was achieved by high-resolution ion exchange (Enrich Q, BioRad #780–0001) or electroelution of the corresponding gel band (GE 200, Hoefer Scientific Instruments, San Francisco, CA, USA). The sequences were confirmed through automated capillary sequencing.

### Animals and drugs

Male albino CD-1 mice and homozygous (σ1R^−/−^) male sigma receptor knockout mice, backcrossed (N10 generation) onto a CD1 albino genetic background (ENVIGO, Milano, Italy) were used in the study. The mice were maintained at 22 °C on a diurnal 12 h light/dark cycle. Procedures involving mice adhered strictly to the guidelines of the European Community for the Care and Use of Laboratory Animals (Council Directive 86/609/EEC) and Spanish law (RD53/2013) regulating animal research. Each group consisted of eight to ten animals, which were used only once. The compounds used were as follows: morphine sulfate (mu-opiod receptor agonist, Merck, Darmstadt, Germany); NMDA (#0114); CBD (#1570); BD1063 (σ1R antagonist #0883); PRE084 (σ1R agonist #0589); (±)-PPCC oxalate (σ1R agonist #3870), 4-IBP (σ1R ligand #0748); WAY100635 maleate (5HT1A receptor antagonist #4380) were obtained from Tocris Bioscience (Bristol, UK). Progesterone (σ1R antagonist P7556) and pregnenolone sulfate (σ1R agonist P162) were obtained from Sigma-Aldrich (Spain). Test drugs were dissolved in saline except CBD and PPCC, which were prepared in a 1:1:18 (v/v/v) mixture of ethanol:Kolliphor EL (#C5135, Sigma-Aldrich): physiological saline, and injected intracerebroventricularly (icv) 30 min before NMDA administration. To facilitate selective and straightforward access to their targets, the compounds were injected (4 μL) into the lateral ventricles of mice as previously described [79]. Animals were lightly anesthetized and injections were performed with a 10 μL Hamilton syringe at a depth of 3 mm at a point of 2 mm lateral and 2 mm caudal from the bregma. The 4 μL were infused at a rate of 1 μL every 5 s. After this the needle was maintained for an additional 10 s. Mice were randomly assigned to each treatment of the selected compounds (power of 80% to detect statistically significant differences). The use of drugs, experimental design and sample size determination were approved by the Ethical Committee for Research of the CSIC (SAF2015–65420 & CAM PROEX 225/14).

### Experimental protocols

#### In vitro interactions between recombinant proteins: Pull-down of recombinant proteins, effect of drugs on σ1R-NR1 interactions

Having demonstrated that the σ1R does not bind to GST (Z02039; GenScript Co., Piscataway, NJ, USA) [26], we assessed the association of GST-free σ1Rs with GST-tagged NMDAR NR1 C-terminal sequence, which was immobilized through covalent attachment to NHS-activated Sepharose 4 fast flow (FF) (GE#17–0906-01; General Electric Healthcare, Spain) according to the manufacturer’s instructions. The recombinant σ1R (100 nM) was incubated either with NHS-blocked Sepharose 4FF (negative control) or with the immobilized NR1 protein fragment in 200 μL of a buffer containing 50 mM Tris-HCl (pH 7.5) and 0.2% CHAPS in the presence of 2.5 mM of CaCl2. In pilot assays, we determined that after 20 min of incubation the NR1-σ1R association was maximal and that, this period of time was also sufficient for the drugs to promote stable changes in their association. Thus, the samples were mixed by rotation for 20 min at RT, and σ1Rs bound to NR1-Sepharose 4FF were recovered by centrifugation and three cycles of washing. The agarose-attached NR1-σ1R complexes were incubated in the presence of increasing concentrations of the drugs under study for 20 min with rotation at room temperature in 300 μL of 50 mM Tris-HCl (pH 7.5), 2.5 mM CaCl2, and 0.2% CHAPS. In this assay, σ1R ligands dissolved in aqueous solutions display calcium- and concentration-dependent activity in altering σ1R-NR1 associations. If an organic solvent, such as DMSO, is required to incorporate the drug under study, i.e., CBD, DMSO must be kept below 1% in the buffer of the assay. Higher concentrations of DMSO stabilize σ1R-NR1 associations and diminish the disruptive effects of σ1R antagonists. Thus, CBD was initially dissolved in 100% DMSO, and through serial dilutions, the concentrations used in the study were obtained with a final DMSO concentration of approximately 1%. Agarose pellets containing the bound proteins were obtained by centrifugation, washed thrice in the presence of 2.5 mM CaCl2, and solubilized in 2× Laemmli buffer, and the content of σ1Rs was addressed by Western blotting.

The detached σ1Rs from the aforementioned procedure were resolved with SDS/polyacrylamide gel electrophoresis (PAGE) in 4–12% Bis-Tris gels (NuPAGE NP0341, Invitrogen, Thermo Fisher Scientific, Spain) with MES SDS running buffer (NuPAGE NP0002, Invitrogen) and then transferred onto 0.2 μm polyvinylidene difluoride (PVDF) membranes (162–0176; Bio-Rad, Madrid, Spain). The anti-σ1R (#42–3300, Invitrogen) diluted in Tris-buffered saline pH 7.7 (TBS) + 0.05% Tween 20 (TTBS) was incubated overnight at 6 °C. The primary antibody was detected using secondary antibodies conjugated to horseradish peroxidase. The western blot images showing antibody binding, were visualized by chemiluminescence (#170–5061; Bio-Rad) and recorded using a ChemiImager IS-5500 (Alpha Innotech, San Leandro, CA).

### Evaluation of antinociception

The response of the animals to nociceptive stimuli was determined by the warm water (52 °C) tail-flick test as previously described [27]. The tail-flick analgesic test applies a thermal noxious stimulus to promote flicking of the mouse’s tail, and opioids given by icv route increase the time elapsed between application of the stimulus and the flick. This response comprises a spinal reflex that is under facilitator drive by the brain stem nociceptive modulating network. Baseline latencies ranged from 1.5 to 2.2 s. A cut-off time of 10 s was used to minimize the risk of tissue damage. Drugs were icv injected and antinociception was assessed at different time intervals thereafter. Saline was likewise administered as a control. Antinociception was expressed as a percentage of the maximum possible effect (MPE = 100 × [test latency-baseline latency]/[cut-off time (10 s)-baseline latency]).

### NMDA-induced seizures

Seizures were induced by injection of NMDA (0.3 and 1 nmol/mouse icv, in a volume of 4 μL sterile saline) as described by others [44]. The dose of 1 nmol NMDA was selected as the minimal dose that reliably induced the appearance of tonic seizures in at least 80% of treated mice. Immediately after injection animals were placed in a transparent box (20x20x30 cm) and were observed for a period of 3 min. The seizure activity consisted of a mild myoclonic phase (immobility, mouth and facial movements, tail extension, circling), rearing (violent movements of the hole body, rearing), wild running (episodes of running with explosive jumps), clonic convulsions (characterized by rigidity of the whole body including limbs flexion/extension), followed by continuous/repetitive seizure activity (tonic seizures) and, in approximately 15–20% of the animals, death. The episode typically began a few seconds after injection and evolved to its maximal intensity in less than 1 min. The results are expressed as the percentage of mice exhibiting the aforementioned signs and the mean latencies of the first body clonus.

### Permanent unilateral middle cerebral artery occlusion (pMCAO) and the determination of infarct size

Focal cerebral ischemia was induced via pMCAO, as described previously [36]. Briefly, mice were anesthetized and a vertical skin incision was made between the left eye and ear under a dissection microscope. After drilling a small hole in the cranium at the level of the distal portion of the middle cerebral artery, the artery was occluded by cauterization. Flow obstruction was visually verified. Animals showing subdural haemorrhages or signs of incorrect surgery were immediately excluded from the study (< 5% in each group). The mice were returned to their cages after surgery, kept at room temperature, and allowed food and water ad libitum. Strong lesion reproducibility was observed. We exclude mice from further studies if excessive bleeding occurs during surgery, mice fail to recover from anaesthesia within 15 min, or haemorrhage was found in the brain during post-mortem examination. The investigator performing the pMCAO surgery was blinded to treatment group. To determine the infarct size 48 h after surgery, animals were euthanized and their brains were removed, after which six 1 mm-thick coronal brain slices (Brain Matrix, WPI, UK) were obtained. The sections were stained with 2,3,5-triphenyltetrazolium chloride (1% TTC, Sigma-Aldrich). Infarct volumes were calculated by sampling each side of the coronal sections with a digital camera (Nikon Coolpix 990, Tokyo, Japan). The extent of unstained infarct area (expressed in mm2) was integrated from the total area as an orthogonal projection.

### Statistical analysis

The data represent the means ± SEM. The Sigmaplot/SigmaStat v.14 package (SPSS Science Software, Erkrath, Germany) was used to generate the graphs, determine parameters (interaction of drugs with σ1R-NR1 complexes), and perform the corresponding statistical analysis. The level of significance was *p* < 0.05. Data were analyzed using one-way ANOVA followed by Dunnett multiple comparisons as appropriated.

## Abbreviations

5HTR: serotonin receptor
CaM: calcium-activated calmodulin
CBD: Cannabidiol
CBR: cannabinoid receptors
GPCR: G-protein-coupled receptor
HINT1: Histidine triad nucleotide-binding protein 1
NMDAR: *N*-methyl-D-aspartate acid receptor
NR1: NMDAR subunit 1
pMCAO: permanent middle cerebral artery occlusion
σ1R: sigma 1 receptor
